# Activation of T Lymphocytes as a Novel Mechanism in Beta1-Adrenergic Receptor Autoantibody-Induced Cardiac Remodeling—Additional Information About TLR9 Involvement

**DOI:** 10.1007/s10557-019-06874-0

**Published:** 2019-03-18

**Authors:** Annekathrin Haberland, Johannes Müller, Katrin Wenzel

**Affiliations:** Berlin Cures GmbH, Berlin, Germany

Du et al. [1] with their recently published interesting investigations on T cell activation caused by beta1-adrenoceptor autoantibodies represent another piece of the puzzle in identifying the entire pathological way of action of these agonistic acting autoantibodies in the pathogenesis of heart disease.

These authors observed that, besides the already well-described activation of the beta1-adrenoceptors directly in the heart cells [2, 3], these autoantibodies additionally activated beta1-adrenoceptors in T lymphocytes [1]. Via the latter, the autoantibodies caused IL-6 release which is, according to their results, a further contributing factor to the pathogenesis of heart failure. Their observation bridges heart failure to an immunological perspective, a perspective which was recently comprehensively reviewed by Alvarez and Briasoulis [4] including current and novel treatment strategies.

There is just one additional aspect we would like to bring up in this context. With respect to initial triggers of heart failure, Eriksson et al., when reporting about the “dendritic cell-induced autoimmune heart failure requires cooperation between adaptive and innate immunity,” investigated the impact of TLR receptors as triggers but also sustainers (“myocarditis relapse”) of heart failure in an animal model as early as 2003 [5]. A work, continued by Oka et al. in 2012, showing “that mitochondrial DNA that escapes from autophagy cell autonomously leads to Toll-like receptor (TLR) 9-mediated inflammatory responses in cardiomyocytes and is capable of inducing myocarditis, and dilated cardiomyopathy” [6].

Both studies together complete a general understanding of the effects of TLR9 on the initiation and subsequent maintenance of heart failure that creates a vicious circle by making TLR9 essential in autoantibody formation [4], which in turn, stimulate receptors in heart cells and T lymphocytes [1].

It would be interesting to know in this context, if beta1-autoantibody activated T cells would be able to act as stimulator of the beta1-autoantibody production, similar to the function of other T cell co-stimulatory factors which again include, e.g., TLR9 ligands [7], sustaining the vicious circle. If so, this would finally offer an explanation for the already observed long-lasting persistence of autoantibody absence after even a one-time removal only, as seen after immunoadsorption [8] and also already at the single application of the autoantibody neutralizer BC 007 (here, observation period of one month) [9]. One treatment would practically be enough to interrupt this cycle. BC 007 is the broadband aptamer for the neutralization of heart failure relevant agonistic acting autoantibodies which attack essential heart and circulatory system–regulating receptors including the beta1-receptor [9, 10]. Independent from an answer to this question, with the antibody neutralizer BC 007, the TLR9 pathway will additionally be counteracted [11]. In cells, overexpressing the human TLR9 receptor and a corresponding reporter protein, BC 007 pre-incubation was able to abrogate agonist-caused TLR9 stimulation (ODN 2006, InvivoGen).

BC 007 is, therefore, able to break into this pathogenic vicious circle via autoantibody neutralization and TLR9 antagonism but would also always be able to act on both pathways alone. With the latter widening, the circle of patients who should benefit from a therapy also possibly including patients showing symptoms and TLR9 overexpression but no or not yet autoantibodies [12].

BC 007, currently under preparation for phase II of the heart failure efficacy testing, has already shown its autoantibody-neutralizing efficacy in humans besides its favorable side effect profile [9]. Derived from the good experiences made with the removal of these autoantibodies via immuoadsorption [2, 13], it is hoped that a huge number of heart failure patients will benefit from this novel causal treatment.
